# The impact of omega-3 polyunsaturated fatty acid supplementation on the incidence of cardiovascular events and complications in peripheral arterial disease: a systematic review and meta-analysis

**DOI:** 10.1186/1471-2261-14-70

**Published:** 2014-05-31

**Authors:** Jennifer E Enns, Azadeh Yeganeh, Ryan Zarychanski, Ahmed M Abou-Setta, Carol Friesen, Peter Zahradka, Carla G Taylor

**Affiliations:** 1 Department of Physiology, University of Manitoba, Winnipeg, Canada; 2 Canadian Centre for Agri-Food Research in Health and Medicine, St. Boniface Research Centre, R2019-351 Taché Ave, Winnipeg R2H 2A6, Canada; 3 Department of Internal Medicine, University of Manitoba, Health Sciences Centre, 820 Sherbrook St, Winnipeg R3T 2N2, Canada; 4 George & Fay Yee Centre for Healthcare Innovation/Winnipeg Regional Health Authority, Health Sciences Centre, University of Manitoba, 820 Sherbrook St, Winnipeg R3T 2N2, Canada; 5 Neil John Maclean Health Sciences Library, University of Manitoba Libraries, 770 Bannatyne Ave, Winnipeg R3E 0W3, Canada; 6 Department of Human Nutritional Sciences, University of Manitoba, Winnipeg, Canada

**Keywords:** Omega-3 polyunsaturated fatty acid, Peripheral arterial disease, Intermittent claudication

## Abstract

**Background:**

Individuals with peripheral arterial disease are at higher risk for cardiovascular events than the general population. While supplementation with omega-3 polyunsaturated fatty acids (PUFA) has been shown to improve vascular function, it remains unclear if supplementation decreases serious clinical outcomes. We conducted a systematic review and meta-analysis to determine whether omega-3 PUFA supplementation reduces the incidence of cardiovascular events and complications in adults with peripheral arterial disease.

**Methods:**

We searched five electronic databases (MEDLINE, EMBASE, CENTRAL, Scopus and the International Clinical Trials Registry Platform) from inception to 6 December 2013 to identify randomized trials of omega-3 PUFA supplementation (from fish or plant oils) that lasted ≥12 weeks in adults with peripheral arterial disease. No language filters were applied. Data on trial design, population characteristics, and health outcomes were extracted. The primary outcome was major adverse cardiac events; secondary outcomes included myocardial infarction, cardiovascular death, stroke, angina, amputation, revascularization procedures, maximum and pain-free walking distance, adverse effects of the intervention, and quality of life. Trial quality was assessed using the Cochrane Risk of Bias tool.

**Results:**

Of 741 citations reviewed, we included five trials enrolling 396 individuals. All included trials were of unclear or high risk of bias. There was no evidence of a protective association of omega-3 PUFA supplementation against major adverse cardiac events (pooled risk ratio 0.73, 95% CI 0.22 to 2.41, *I*^*2*^ 75%, 2 trials, 288 individuals) or other serious clinical outcomes. Adverse events and compliance were poorly reported.

**Conclusions:**

Our results showed that insufficient evidence exists to suggest a beneficial effect of omega-3 PUFA supplementation in adults with peripheral arterial disease with regard to cardiovascular events and other serious clinical outcomes.

## Background

Peripheral arterial disease is an atherosclerosis-induced blockage of non-coronary and non-cerebral arteries. Individuals diagnosed with peripheral arterial disease most often present with ischemic pain in the lower leg following exercise, and as the disease progresses, pain may also occur at rest [1]. The management of peripheral arterial disease includes therapies that reduce atherosclerotic disease progression and cardiovascular events, such as vasoactive drugs, statins, smoking cessation therapy, exercise, and in severe cases, angioplasty or bypass surgery [2]. Risk factor modification unfortunately has little effect on the primary symptom of peripheral arterial disease, intermittent claudication [2]. Peripheral arterial disease is estimated to occur in 5.9% of people older than 40 years, corresponding to 7.2 million affected individuals in the United States alone [3]. While nearly 60% of all individuals with peripheral arterial disease are asymptomatic [4], they are still at high risk for coronary heart disease due to the underlying presence of atherosclerosis. Cardiac events are the most common cause of death in persons with peripheral arterial disease [5].

Omega-3 polyunsaturated fatty acids (PUFA) are a group of dietary fats obtained from fish and plant oils that are reported to have a protective role in coronary heart disease and other cardiovascular complications [6]. Omega-3 PUFA may exert beneficial effects in cardiovascular disease by lowering hepatic triglyceride production and increasing clearance from the circulation [7], and by incorporating into phospholipids in cell membranes, thereby reducing the availability of substrates for the production of pro-inflammatory molecules [8]. Systematic reviews of omega-3 PUFA supplementation cohort studies and randomized controlled trials have focused primarily on the marine-sourced omega-3 PUFA eicosapentaenoic acid (EPA) and docosahexaenoic acid (DHA). Many of these reviews have demonstrated reductions in cardiovascular mortality in populations with and without established cardiovascular disease [6,9-14], while others have shown mixed results [15-18] or no benefits [19-21] following marine omega-3 PUFA supplementation. However, little is known about the role of the plant-based omega-3 PUFA alpha-linolenic acid (ALA) in cardiovascular disease, particularly in the high-risk population of individuals with peripheral arterial disease.

Given the high incidence of cardiovascular events in individuals with peripheral arterial disease [22] and the demonstrated benefits of omega-3 PUFA in individuals with cardiovascular disease [6], several clinical trials of omega-3 PUFA supplementation have been conducted in the peripheral arterial disease population. While some of these trials have shown improvements in vascular function [23-25] or inflammatory status [26], it is unclear if supplementation decreases the incidence of major adverse cardiovascular events and other relevant clinical outcomes. The objective of this systematic review was to determine whether dietary supplementation with fish and/or plant-based omega-3 PUFA reduces the incidence of cardiac events and complications in the high-risk population of peripheral arterial disease patients.

## Methods

Using an *a priori* published protocol [27], we conducted a systematic review using methodological approaches outlined in the *Cochrane Handbook for Systematic Reviewers*[28] and reported according to the Preferred Reporting Items for Systematic Reviews and Meta-Analyses (PRISMA) criteria [29].

### Data sources and searches

We searched the following bibliographic databases from inception to Dec 6, 2013: PubMed/MEDLINE (National Library of Science), EMBASE (Ovid), CENTRAL (Cochrane library - Wiley), followed by forward searching for key articles in Scopus. To identify ongoing and unpublished trials, we searched the World Health Organization’s International Clinical Trials Registry Platform. We supplemented electronic searches by hand-searching the bibliographies of included trials, and relevant narrative and systematic reviews. Our search strategy used both controlled vocabulary and free text, and searches in MEDLINE and EMBASE were combined with a high-sensitivity filter for randomized controlled trials [28]. The detailed search strategy for MEDLINE is included in Table 1.

**Table 1:** Search strategy

1.	(omega 3 fatty acid OR omega-3 fatty acid OR n 3 fatty acid OR n-3 fatty acid OR n-3 polyunsaturated fatty acid)
2.	(dietary supplement OR dietary supplementation OR dietary fat)
3.	(flax OR flaxseed OR flaxseed oil OR linseed oil)
4.	(fish OR fish oil OR fatty fish OR marine)
5.	(canola OR canola oil OR rapeseed oil)
6.	(dietary supplements[MeSH Terms]) OR (dietary fats[MeSH Terms]) OR (flax[MeSH Terms]) OR (linseed oil[MeSH Terms]) OR (fishes[MeSH Terms]) OR (fish oils[MeSH Terms])
7.	(eicosapentaenoic acid OR EPA)
8.	(docosahexaenoic acids OR DHA)
9.	(alpha-linolenic acid OR ALA)
10.	(fatty acids, omega-3[MeSH Terms]) OR (eicosapentaenoic acid[MeSH Terms]) OR (docosahexaenoic acids[MeSH Terms]) OR (alpha-linolenic acid[MeSH Terms])
11.	#1 OR #2 OR #3 OR #4 OR #5 OR #6 OR #7 OR #8 OR #9 OR #10
12.	(peripheral arterial disease OR peripheral artery disease OR peripheral arterial diseases OR peripheral artery diseases)
13.	(peripheral vascular disease OR peripheral vascular diseases OR peripheral angiopathy OR peripheral angiopathies)
14.	(ankle brachial index OR ankle-brachial index OR ankle brachial indices OR ankle-brachial indices OR intermittent claudication)
15.	(peripheral arterial disease[MeSH Terms]) OR (peripheral vascular diseases[MeSH Terms]) OR (ankle-brachial index[MeSH Terms]) OR (intermittent claudication[MeSH Terms])
16.	#12 OR #13 OR #14 OR #15
17.	(randomized controlled trial[pt] OR controlled clinical trial[pt] OR randomized[tiab] OR placebo[tiab] OR drug therapy[sh] OR randomly[tiab] OR trial[tiab] OR groups[tiab]) NOT (animals[mh] NOT humans[mh])
18.	#11 AND #16 AND #17

### Study selection

To identify a population at high risk for cardiovascular events, we included only randomized trials of adults aged 40 years or older with established peripheral arterial disease, which was diagnosed by the presence of stable intermittent claudication and/or an ankle-brachial index ≤ 0.9. The intervention period was required to be at least 12 weeks in duration to allow the intervention to impact cardiovascular function. The minimum intervention duration of 12 weeks was chosen based on studies of the appearance of dietary fatty acids in the plasma and blood cell plasma membranes (which are considered robust markers of omega-3 PUFA consumption), demonstrating that plasma membrane fatty acid composition changes are apparent within two weeks of a dietary fatty acid intervention [30]. Thus, it is feasible that downstream physiological effects of the diet could be demonstrated within 12 weeks. We included interventions with any omega-3 PUFA in diet or supplement form where the dose per day was reported and with appropriate comparators (i.e. placebo, omega-3 PUFA deficient diet or usual diet). We used a two-stage process for trial screening and selection employing standardized and piloted screening forms. Two reviewers independently screened the titles and abstracts of the search results to determine whether each citation met the inclusion criteria. Trials published in languages other than English were translated, and the full text of citations classified as *include* or *unclear* were independently reviewed with reference to the predetermined inclusion and exclusion criteria. Discrepancies between reviewers were resolved through consensus or by discussion with a third reviewer, as required.

### Data extraction and quality assessment

From each trial, two reviewers independently abstracted population characteristics (including age, body mass index, and diagnosis of peripheral arterial disease), number of participants, trial duration and follow-up, intervention design (including type and dose of omega-3 PUFA, comparators, and co-interventions), clinical health outcomes (incidence of major adverse cardiac events, myocardial infarction, cardiovascular death, angina, and stroke, amputation, symptom-driven revascularization procedures (e.g. bypass surgery), maximum and pain-free walking distance, and quality of life), and adverse effects. Any disagreements were resolved through consensus. Where data were incompletely or imprecisely reported, we contacted study authors for clarification. We assessed the internal validity of the included trials using the Cochrane Collaboration Risk of Bias Tool [31]. This tool consists of six domains, each of which is rated “low risk,” “unclear risk,” or “high risk.” If one or more individual domains were assessed as having a high risk of bias, the overall rating was a high risk of bias. We considered the overall risk of bias low only if all components were rated as having a low risk of bias. We rated the risk of bias for all other trials as unclear. Information regarding methodological quality was used to guide subgroup analyses and to explore sources of heterogeneity.

### Data synthesis and analysis

We conducted meta-analyses of the data from included trials using Review Manager (Version 5.2, The Cochrane Collaboration, Copenhagen, Denmark). Pooled binary data were weighted using the Mantel-Haenszel method, and presented as risk ratios (or Peto odds ratios for rare events) with 95% confidence intervals (CI) [32]. Pooled continuous data were weighted by the inverse of variance and expressed as a weighted mean difference with 95% CI. We explored and quantified statistical heterogeneity of the data using the I-squared test [33]. We assessed publication bias by viewing the overlap of confidence intervals and using funnel plot techniques. For the primary outcome of major adverse cardiac events, we performed the following *a priori* subgroup analyses: short versus extended duration of intervention, high versus low omega-3 PUFA dose (g/day), type of omega-3 PUFA (EPA, DHA, ALA, or a combination of these), supplements or capsules vs. dietary sources of omega-3 PUFA, and the duration of follow-up. Final subgroup analyses were limited by the number of trials included and the availability of reported outcomes and covariates.

## Results

### Characteristics of trial populations and interventions

**Figure 1:**
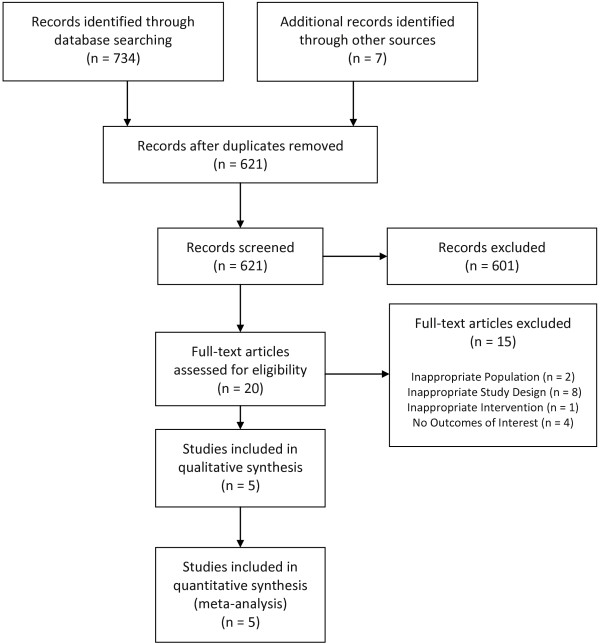
**PRISMA flow diagram.** Summary of the evidence search and selection.

Of 741 citations identified from electronic and hand-searches, we included 5 unique trials enrolling a total of 396 individuals (Figure 1; Table 2). All were single-centre trials published between 1990 and 2010. All trials were adjudicated to be of high (3/5) [34-36] or unclear (2/5) [37,38] risk of bias (Table 3). Due to the small number of trials included, formal assessment of reporting bias (e.g., by using a funnel plot) was not possible.

**Table 2:** Patient populations and study characteristics

**Study**	**Population**					**Intervention**			
	**Age (years)**	**BMI (kg/m**^**2**^**)**	**Diagnosis of PAD**	**Medication/therapy**	**Co-morbidities**	**Omega-3 PUFA (g/day)**	**Comparator (g/day)**	**Co-intervention**	**Duration**
Gans 1990 [37]	66.1 ± 8.2	NR	Treadmill testing for IC classification; IC stable for ≥ 1 yr	Excluded patients on lipid-lowering or platelet-active drugs	None reported	EPA (1.8) + DHA (1.2)	Corn oil (3.0)	None	16 wk
Leng 1998 [34]	65.7 ± 7.1	26.7 ± 4.2	Edinburgh Claudication Questionnaire; IC stable for ≥ 6 mth; ABI < 0.9	Included patients taking aspirin	Excluded patients with critical ischemia, previous or impending surgery, unstable angina or MI, or severe concurrent illnesses	EPA (0.18-0.27)	Sunflower oil (2.0 – 3.0)	Gamma-linolenic acid	2 yr
Carrero 2005 [35]	64.0 ± 9.0	27.9 ± 3.9	Presence of IC; ABI < 0.7	Excluded patients eligible for vascular surgery; excluded patients taking statins	Excluded patients with history of cardiac events or with endocrine or metabolic disturbances. Included patients who were smokers, had T2D or hypertension	EPA (0.2) + DHA (0.13) + ALA (0.06)	Placebo (dairy product)	Vitamins B_6_ and E, folate, oleic acid	1 yr
Carrero 2006 [36]	65.5 ± 9.5	27.7 ± 3.4	Presence of IC; ABI < 0.7	Excluded patients eligible for vascular surgery; excluded patients taking statins	Excluded patients with history of cardiac events or with endocrine or metabolic disturbances. Included patients who were smokers, had T2D or hypertension	EPA (0.2) + DHA (0.13) + ALA (0.06)	Placebo (dairy product)	Vitamins B_6_ and E, folate, oleic acid	1 yr
Ishikawa 2010 [38]	65.2 ± 7.4	23.3 ± 2.8	Presence of IC; physical findings (e.g. ulcer) and ABI	All patients included were taking statins	Hyperlipidemia (total serum cholesterol)	EPA (1.8)	No treatment	Simvastatin or Pravastatin	up to 5 yr

Data are presented as mean ± SD. ABI: ankle-brachial index; ALA: alpha-linolenic acid; BMI: body mass index; DHA: docosahexaenoic acid; EPA: eicosapentaenoic acid; IC: intermittent claudication; MI: myocardial infarction; T2D: type 2 diabetes.

**Table 3:** Risk of bias assessment

**Study**	**Selection bias**		**Performance bias**	**Attrition bias**	**Reporting bias**	**Other sources of bias**	**Overall risk of bias**
	**Random sequence generation**	**Allocation concealment**	**Blinding of participants and personnel**	**Incomplete outcome data**	**Selective reporting**		
Gans 1990 [37]	Low risk	Low risk	Unclear risk	Unclear risk	Unclear risk	Low risk	**Unclear risk**
Leng 1998 [34]	Low risk	Low risk	Low risk	High risk^¶^	Unclear risk	Unclear risk*	**High risk**
Carrero 2005 [35]	Low risk	Unclear risk	Low risk	Unclear risk	Unclear risk	High risk^§^	**High risk**
Carrero 2006 [36]	Low risk	Unclear risk	Low risk	Unclear risk	Unclear risk	High risk^§^	**High risk**
Ishikawa 2010 [38]	Low risk	Unclear risk	Unclear risk	Unclear risk	Unclear risk	Low risk	**Unclear risk**

*Intervention group received gamma-linolenic acid, which was not supplied in control.

^§^Intervention group received vitamins B_6_ and E, folate and oleic acid, which were not supplied in control.

^¶^Drop-out rate was > 30%.

Four trials were conducted in Europe [34-37] and one in Japan [38]. One trial recruited individuals who were diagnosed with peripheral arterial disease and were also hypercholesterolemic and on statin therapy [38]. One trial included individuals who previously had angina or a myocardial infarction [34], while the rest excluded individuals with a recent history of cardiovascular/cerebrovascular events or revascularization surgery. Three trials [34,37,38] studied capsules containing varying doses of EPA alone or EPA and DHA combined, compared to capsules of sunflower oil [34] or corn oil [37], or no treatment [38]. The intervention in the remaining two trials [35,36] studied a specially developed dairy product enriched with EPA, DHA and ALA, as well as vitamins A, B_6_, D and E, folate and oleic acid, compared to a control group who received a product identical in appearance enriched only with vitamins A and D. None of the trials had follow-up periods beyond the duration of the intervention.

### Risk of bias in included trials

While all trials had random sequence generation (5/5), fewer reported adequate allocation concealment (2/5) [34,37] and blinding of outcome assessment (3/5) [34-36], and all trials were subject to unclear or high risk of attrition bias due to incomplete outcome data following substantial loss to follow-up or poor reporting of outcomes (5/5) (Table 3). The presence of co-intervention nutrients in 3/5 trials [34-36] did not allow proper controlling for omega-3 PUFA effect.

### Major adverse cardiac events

**Figure 2:**
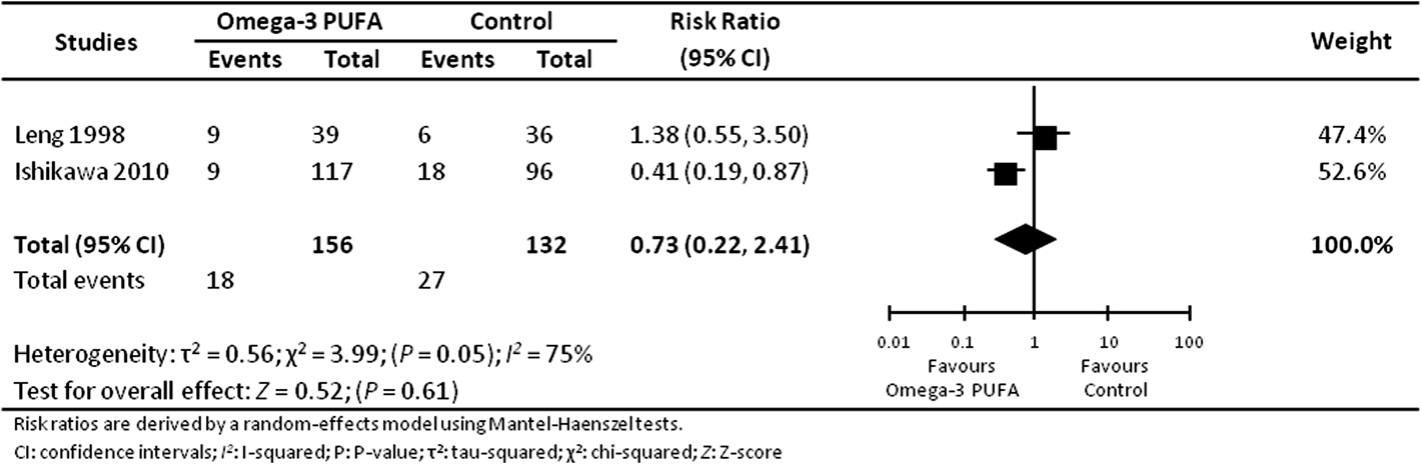
Effect of omega-3 PUFA supplementation on major adverse cardiac events in individuals with peripheral arterial disease.

Two trials reported the incidence of major adverse cardiac events following an intervention with EPA only [34,38]. The risk ratio for major adverse cardiac events was not significantly decreased with supplementation of EPA (risk ratio 0.73; 95% CI 0.22 to 2.41; *I*^*2*^ 75%; 2 trials [34,38]; 288 individuals; Figure 2). High statistical heterogeneity observed may relate to differences in the trial populations (Japanese vs. British, hyper- vs. normo-cholesterolemic), the ten-fold difference in daily dose of EPA (1.8 g vs. 0.18 g), the comparator in the control group (no treatment vs. sunflower oil), the co-intervention (statins vs. gamma-linolenic acid), or the length of the intervention (five vs. two years). In one of the trials (213 individuals), the risk ratio for major adverse cardiac events associated with 1.8 g/day EPA compared to no treatment for 5 years was 0.41; 95% CI 0.19 to 0.87 [38]. In the second trial (75 individuals) comparing 0.18 g/day EPA to sunflower oil for 2 years, the risk ratio for major adverse cardiac events was 1.38; 95% CI 0.55 to 3.50 [34].

### Secondary clinical outcomes

Two trials with a total of 288 individuals reported the incidence of myocardial infarction, cardiovascular death and revascularization procedures (Table 4) [34,38]. The pooled Peto odds ratios for myocardial infarction (Peto odds ratio 0.64; 95% CI 0.22 to 1.88), cardiovascular death (Peto odds ratio 0.60, 95% CI 0.19 to 1.90) and the pooled risk ratio for revascularization surgery (risk ratio 0.81, 95% CI, 0.13 to 4.91) demonstrated no significant difference between the EPA intervention group and control (no treatment or sunflower oil).

**Table 4:** Secondary outcome measures

**Outcome**	**No. of trials**	**No. of events/total patients in cohort**		**Effect estimate (95% CI)**	***I***^***2***^
		**Omega-3 PUFA**	**Control**		
Myocardial Infarction (Leng 1998 [34], Ishikawa 2010 [38])	2	6/156	8/132	Peto OR, 0.64 (0.22, 1.88)	0%
Cardiovascular Death (Leng 1998 [34], Ishikawa 2010 [38])	2	5/156	7/132	Peto OR, 0.60 (0.19, 1.90)	0%
Stroke (Leng 1998 [34])	1	3/60	1/60	Peto OR, 2.79 (0.38, 20.31)	NE
Angina (Ishikawa 2010 [38])	1	5/117	8/106	RR, 0.57 (0.19, 1.68)	NE
Adverse Effects					
All Adverse Events (Gans 1990 [37], Leng 1998 [34])	2	17/76	21/76	RR 0.81 (0.48, 1.38)	NE
Gastrointestinal Upset (Leng 1998 [34])	1	30/60	19/60	RR 1.58 (1.01, 2.48)	NE
Revascularization Surgery (Leng 1998 [34], Ishikawa 2010 [38])	2	9/156	13/132	RR, 0.81 (0.13, 4.91)	59%
Amputation (Leng 1998 [34])	1	0/60	1/60	Peto OR, 0.14 (0.00, 6.82)	NE
Pain-Free Walking Distance (Gans 1990 [37], Leng 1998 [34], Carrero 2005 [35], Carrero 2006 [36])	4	95	88	MD, 115.40 (-42.24, 273.05)	89%
Maximum Walking Distance (Gans 1990 [37])	1	16	16	MD, -26.00 (-71.92, 19.92)	NE

Walking distances (mean differences) are expressed in metres.

CI: confidence interval; MD: mean difference; NE: not estimable; OR: odds ratio; RR: risk ratio; *I*^2^: I-squared.

**Figure 3:**
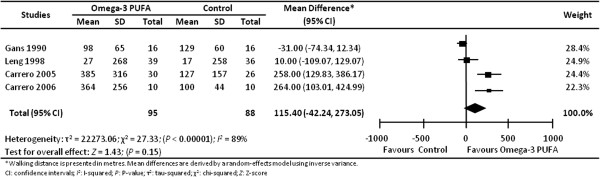
Effect of omega-3 PUFA supplementation on pain-free walking distance in individuals with peripheral arterial disease.

Four trials [34-37] with a total of 187 individuals reported pain-free walking distance (metres), an indicator of intermittent claudication (Figure 3). We observed considerable heterogeneity and no statistically significant differences in pain-free walking distance associated with omega-3 PUFA supplementation (mean difference 115.40 metres; 95% CI -42.24 to 273.05; *I*^2^ 89%). None of the trials reported on quality of life. The remaining secondary outcomes (stroke, angina, amputation and maximum walking distance) were each reported by only one trial and were not found to be statistically different with omega-3 PUFA intervention (Table 4). Gastrointestinal upset, reported in a single trial [34], was more frequent with EPA treatment (risk ratio 1.58; 95% CI 1.01 to 2.48; 60 individuals).

### Subgroup analyses

Limitations of the data (i.e. the number of trials included, and the availability of appropriate outcome data and covariates reported) precluded most subgroup analyses. Comparisons of the effects of omega-3 PUFA intervention length, dose and type of omega-3 PUFA on pain-free walking distance did not reach significance, and statistical heterogeneity remained high (Table 5).

**Table 5:** Subgroup analysis: omega-3 PUFA and pain-free walking distance

**Subgroup**	**No. of trials**	**Total patients in cohort**		**Effect estimate (95% CI)**	***I***^***2***^
		**Omega-3 PUFA**	**Control**		
***Effect of Intervention Duration on PFWD***					
Less than 6 months (Gans 1990 [37])	1	16	16	MD, -31.00 (-74.34, 12.34)	NE
6 months or longer (Leng 1998 [34], Carrero 2005 [35], Carrero 2006 [36])	3	79	72	MD, 172.98 (-0.82, 346.77)	80%
***Effect of Omega-3 PUFA Dose on PFWD***					
More than 0.3 g/day (Gans 1990 [37])	1	16	16	MD, -31.00 (-74.34, 12.34)	NE
Less than or equal to 0.3 g/day (Leng 1998 [34], Carrero 2005 [36], Carrero 2006 [37])	3	79	72	MD, 172.98 (-0.82, 346.77)	80%
***Effect of Omega-3 PUFA Type on PFWD***					
EPA + DHA + ALA (Carrero 2005 [35], Carrero 2006 [36])	2	40	36	MD, 260.33 (160.05, 360.60)	0%
EPA + DHA (Gans 1990 [37])	1	16	16	MD, -31.00 (-74.34, 12.34)	NE
EPA (Leng 1998 [34])	1	39	36	MD, 10.00 (-109.07, 129.07)	NE

ALA, alpha-linolenic acid; CI, confidence intervals; DHA, docosahexaenoic acid; EPA, eicosapentaenoic acid; MD, mean difference; PFWD, pain-free walking distance; *I*^2^, I-squared.

## Discussion

In this systematic review and meta-analysis of omega-3 PUFA in adults with peripheral arterial disease, we found no evidence to suggest a protective association between omega-3 PUFA supplementation and clinical cardiovascular outcomes, including myocardial infarction, cardiovascular death, angina, stroke, amputation, revascularization, pain-free walking distance or quality of life. One trial indicated that omega-3 PUFA intake may be associated with increased gastrointestinal side effects.

The effect of omega-3 PUFA supplementation in cardiovascular disease is controversial. Some reviews and meta-analyses have demonstrated reductions in adverse events with both plant-based ALA and marine-sourced EPA and DHA supplementation in cardiovascular [9-14,39] and cerebrovascular disease [40], and marine omega-3 PUFA may be effective in preventing atrial fibrillation after cardiac surgery [41,42], although this finding remains controversial [43]. Other reviews have demonstrated mixed [6,15-18] or no benefits [19-21] following marine and/or plant-based omega-3 PUFA supplementation. In peripheral arterial disease populations, supplementation with EPA and DHA has been shown to significantly reduce measures of arterial stiffness in several cohorts, including healthy and overweight individuals, and individuals with cardiovascular risk factors, type 2 diabetes, or hypertension [23-25,44]. Even so, adequate long-term data on serious adverse events in peripheral arterial disease populations is lacking.

In this review, the small number of trials available and the lack of uniformity in population and design of the included trials contributed to the relatively high statistical heterogeneity for some outcomes, and thus caution must be exercised in interpreting these results. Some of the key differences among the included trials included geographic differences (e.g., the Japanese population’s omega-3 PUFA consumption is up to 15 times greater than in Western countries [45,46]), variable intervention durations (from 16 weeks up to several years), differences in doses of omega-3 PUFA (doses of 0.13 g/day DHA and 0.2 g/day EPA are probably too low to have an effect), and concomitant statin and vitamin therapy in some trials (which may have provided beneficial effects on cardiovascular function [47]). Finally, the potential influence of the trial sponsor should also not go unnoticed as trials conducted by Carrero et al. [35,36] were sponsored by the manufacturers of omega-3 PUFA.

Only two of the included trials [37,38] supplied therapeutic omega-3 PUFA doses of >1 g/day, according to the recommendations from the American Heart Association and the World Health Organization [48,49]. Subgroup analyses suggest that a longer period of treatment (6 months or longer) even at a low omega-3 PUFA dose may improve walking distance. This exploratory finding must be confirmed in future studies. It should also be noted that only two trials [34,37] reported on adverse side effects: while the trial by Gans et al. [37] reported no significant adverse effects, half of the individuals in the omega-3 PUFA group in the trial conducted by Leng et al. [34] experienced gastrointestinal upset. Growing recognition for nutritional components and supplements as therapeutic agents highlights the need for thorough safety testing during clinical trials.

The internal validity of the trials was often unclear due to underreporting of methods, which potentially could have biased the results. All of the trials rated poorly in the risk of bias assessment, mostly due to a lack of detailed reporting and uncertainty around the true effect of omega-3 PUFA alone (Table 3). If meaningful and reliable results are to be obtained from future randomized controlled trials, substantial improvements in trial design must be made to reduce the risk of bias, ensure sufficient statistical power, and test for the effect of omega-3 PUFA without confounding factors.

Strengths of this review include the broad bibliographic screening of multiple citation databases and trial registries, and rigorous testing for bias. We focused on patient-centred outcomes and evaluated efficacy in the context of relevant side effects. We used an *a priori* published protocol and followed established methodological guidelines in the conduct and reporting of this review. It should be noted that the results of the recent update to the Cochrane review on omega-3 PUFA supplementation in individuals with intermittent claudication [50] are in agreement with our findings, with very little indication for recommending omega-3 PUFA as a therapeutic approach in individuals with peripheral arterial disease. However, the focus of the Cochrane review was primarily on marine omega-3 PUFA, and the presence of ALA in some of the interventions was not acknowledged.

Limitations of this review include the restricted amount of clinical outcome data available for pooling among trials, which may have affected the overall findings. We chose to pool data from trials that were variable in intervention design and population.

## Conclusions

In individuals with peripheral arterial disease, insufficient evidence exists to suggest a beneficial effect of omega-3 PUFA supplementation with regard to major adverse cardiac events, need for revascularization or amputation, pain-free walking disease, or quality of life. Rigorously designed randomized trials powered to detect these clinically relevant outcomes are needed to establish the efficacy and safety of omega-3 PUFA in individuals with peripheral arterial disease.

## Abbreviations

ABI: Ankle-brachial index; ALA: Alpha-linolenic acid; BMI: Body mass index; CI: Confidence interval; DHA: Docosahexaenoic acid; EPA: Eicosapentaenoic acid; *I*^*2*^: I-squared; IC: Intermittent claudication; MD: Mean difference; MI: Myocardial infarction; NE: Not estimable; OR: Odds ratio; PFWD: Pain-free walking distance; PUFA: Polyunsaturated fatty acid; T2D: Type 2 diabetes.

## Competing interests

No funding was obtained specifically for this systematic review. JE is supported by a Food Advancement through Science and Training (FAST) scholarship through the National Sciences and Engineering Research Council’s Collaborative Research and Training Experience (CREATE) program. The authors declare no conflicts of interest.

## Authors’ contributions

An expert in vascular and metabolic disease (JE) led the review team, which included a panel of content experts from multiple fields: vascular physiology (PZ), lipid nutrition (CGT), and research methodology (RZ, AMAS). All authors assisted in formulating the review question, CF assisted with developing the search strategies, RZ and AMAS guided the review methods, and AY assisted with screening and data extraction throughout the review process. JE drafted the manuscript with input from all other authors. All authors read and approved the final manuscript.

## Pre-publication history

The pre-publication history for this paper can be accessed here:

http://www.biomedcentral.com/1471-2261/14/70/prepub
